# The IL-17 Family of Cytokines in Psoriasis: IL-17A and Beyond

**DOI:** 10.3389/fimmu.2018.01682

**Published:** 2018-08-02

**Authors:** Nicolo Costantino Brembilla, Luisa Senra, Wolf-Henning Boehncke

**Affiliations:** ^1^Department of Pathology and Immunology, Faculty of Medicine, University of Geneva, Geneva, Switzerland; ^2^Division of Dermatology and Venereology, Geneva University Hospitals, Geneva, Switzerland

**Keywords:** psoriasis, IL-17 family, IL-25, IL-17E, interleukin, comorbidities, Th17 cells, IL-17A

## Abstract

Psoriasis is a frequent chronic inflammatory skin disease, nowadays considered a major global health problem. Several new drugs, targeting the IL-23/IL-17A pathway, have been recently licensed or are in clinical development. These therapies represent a major improvement of the way in which psoriasis is managed, since they show an unprecedented efficacy on skin symptoms of psoriasis. This has been made possible, thanks to an increasingly more accurate pathogenic view of psoriasis. Today, the belief that Th17 cells mediate psoriasis is moving to the concept of psoriasis as an IL-17A-driven disease. New questions arise at the horizon, given that IL-17A is part of a newly described family of cytokines, which has five distinct homologous: IL-17B, IL-17C, IL-17D, IL-17E, also known as IL-25 and IL-17F. IL-17 family cytokines elicit similar effects in target cells, but simultaneously trigger different and sometimes opposite functions in a tissue-specific manner. This is complicated by the fact that IL-17 cytokines show a high capacity of synergisms with other inflammatory stimuli. In this review, we will summarize the current knowledge around the cytokines belonging to the IL-17 family in relation to skin inflammation in general and psoriasis in particular, and discuss possible clinical implications. A comprehensive understanding of the different roles played by the IL-17 cytokines is crucial to appreciate current and developing therapies and to allow an effective pathogenesis- and mechanisms-driven drug design.

## Introduction

Psoriasis is a frequent, chronic, non-communicable inflammatory skin disease, for which there is no clear cause or cure. Psoriasis affects people of all countries and of all ages. The disease manifests as well-defined, red, scaly plaques, appearing with a chronic-recurrent course at preferential sites such as elbows, knees, and scalp. Individuals with psoriasis are at an increased risk of developing other chronic and serious diseases, including psoriatic arthritis, metabolic syndrome, cardiovascular diseases, and depression (1–3). The negative impact of psoriasis on people’s lives can be immense. Disfiguration and disability are common aspects experienced by patients, who are in addition challenged by emotional and social burden caused by psoriasis stigmatization. In 2014, the Executive Board of the Word Health Organization became spokesman of more than 100 million people affected worldwide, approving a resolution to raise awareness of psoriasis as a major global health problem (4).

Psoriatic hallmark features include profound modification of the epidermis, such as hyperproliferation and altered differentiation of keratinocytes, concurrent with the presence of a prominent inflammatory infiltrate and neo-angiogenesis. T cells, along with innate immune cells, are thought to produce a key effector cytokine, IL-17A, which then triggers the epidermal modification (1). The emergence of a detailed pathogenic concept in the last decade has fueled the development of targeted therapies. Thus, treatments have moved from broad immunosuppression to interference with T cells and the IL-23/IL-17 pathway, and nowadays to downstream effector molecules such as IL-17A and its cell targets (5).

IL-17A was initially thought to be a “unique” cytokine, exclusively produced by T cells in psoriasis. To date, it is evident that many other cells contribute to the bulk of IL-17A found in the diseased skin, and that many isoforms of IL-17 may participate to psoriasis. These data open a new scenario for innovative therapeutic interventions, as our knowledge of the pathophysiology of psoriasis becomes more precise. This review will briefly debate the role of IL-17A as key effector molecule, retracing the key discoveries that led to the current understanding of psoriasis as an IL-17A driven disease. This will lead to an in-depth discussion of the IL-17 family of cytokines and the contribution of IL-17 isoforms other than IL-17A to psoriasis manifestations in the skin and its comorbidities. Finally, the clinical implications will be addressed.

## Psoriasis: An IL-17A-Driven Disease

In the 1960, psoriasis was thought to be a primary disease of the epidermis caused by hyperproliferative keratinocytes. The involvement of the immune system became apparent only in the 1980, when lymphocyte-targeted therapies were proven to be an effective way to treat the disease (6, 7). The pathogenesis of psoriasis was initially proposed to rely on Th1 responses, based on the identification of elevated expression of Th1 cytokines, such as IFNγ, TNFα, and IL-12, in the lesion (8). In the wake of these results, a monoclonal antibody (ustekinumab) designed to block the p40 subunit of IL-12, key factor in Th1 cell commitment, was developed. This antibody showed the highest therapeutical efficacy ever observed at that time. Concomitant to ustekinumab generation, a second cytokine, named IL-23, was found to contain the identical p40 subunit (9). The “unwanted” blockade of this latter cytokine turned out to be the more relevant mechanism of action in the context of treating psoriasis.

In the mid 2000s, IL-23 was shown to induce the production of IL-17 by activated T lymphocytes, later named Th17 cells (10, 11). These cells, which express RORγt as master transcription factor, have limited inherent pathogenicity and promote mucosal defense, whereas exposure to IL-23 turns them into autoimmune-associated inflammatory cells (12). The involvement of IL-23 in psoriasis was supported by its ability to induce psoriasiform characteristics in a preclinical model of intradermal administration (13); a phenotype linked to the infiltration of IL-22- and IL-17A-producing T cells (14). Th17 cells became thus the center of extensive research, and the hallmark cytokine IL-17A was identified as a novel key effector pathogenic factor in psoriasis.

Genome-wide association studies also confirm the role of the immune system in the pathogenesis of psoriasis, with the HLA-Cw6 allele accounting for almost 50% of the disease heritability. Variations in loci containing genes involved in the IL-23/Th17 signaling are frequently observed and suggest the particular involvement of Th17 cells: these include genes upstream of the IL-17 expression, such as IL-23R and IL-12B, or downstream the IL-17 receptor, such as STAT3 and Act1 (15). No variants in the IL-17A gene itself was shown to predispose to psoriasis so far, whereas the IL-17RA allele rs4819554 was recently associated with risk of developing psoriasis in a Spanish cohort (16).

The central role of IL-17A in the pathophysiology of psoriasis has recently been reviewed elsewhere (17) and will only be briefly described here. IL-17A mainly acts on non-hematopoietic cells, particularly epithelial cells, and consistently participates in protective immunity at boundary tissues. With regard to the skin, IL-17A leads to increased proliferation and aberrant differentiation of keratinocytes (18) and contributes to skin barrier disruption by downregulating the expression of molecules involved in keratinocyte differentiation, such as fillagrin (19). In addition, IL-17A participates in generating and amplifying the inflammatory network by promoting the release of antimicrobial peptides and proinflammatory cytokines/chemokines (20, 21). The factors induced by IL-17A are poised toward the activation of a neutrophil/Th17 cell-dependent immune response. These include IL-8, a potent neutrophil chemoattractant; G-CSF, a survival factor for neutrophils; CCL20 that favors Th17 cell recruitment; and the key Th17 polarizing cytokines IL-1β and IL-6. In addition, IL-17A directly contributes to leukocyte migration and tissue remodeling by promoting the secretion of metalloproteases. To note, IL-17A synergizes with and potentiates the effects of many other inflammatory mediators, possibly *via* stabilization of target mRNA. IL-10 and IL-1 family members, as well as type-I cytokines, such as TNFα, are the most relevant factors in this regard (22–24). The genes synergistically upregulated by TNFα and IL-17A in keratinocytes were shown to mimic the gene signature observed in the lesional skin, underling the importance of these integrative responses (23). Similarly, IL-17A, together with TNF and IL-22, were reported to upregulate the expression of the IL-1 like family member IL-36, which in turn was found to augment the function of Th17 cytokines, revealing the existence of a feedback loop between Th17 and IL-36 cytokines (24). These cytokine networks may also be of particular importance to distinguish different forms of psoriasis: inactivating mutation of the IL36RN gene, which encodes the IL-36 receptor antagonist, have been particularly associated with generalized pustular psoriasis (25). The importance of IL-17A and its interaction with other cytokines has also extensively been proved in murine models of psoriasiform inflammation, through the use of deficient mice and in neutralizing experiments. Finally, the first biologics following ustekinumab that entered the market of anti-psoriatics were specific anti IL-17A antibodies, namely secukinumab and ixekizumab (26, 27). Stressing the importance of IL-17A, these therapies represent the most effective approach to treat psoriasis so far.

The effects of IL-17A are not limited to keratinocytes and encompass several other cells, including endothelial cells, fibroblasts, chondrocytes, and synovial cells. IL-17A is clearly of major importance also in the context of psoriasis-associated comorbidity, namely, psoriatic arthritis and cardiovascular disease/atherosclerosis, as highlighted elsewhere (28, 29) and summarized in Table 1.

**Table 1:** Overview of the role exerted by IL-17A in inflammation.

Skin inflammation – *Human/patient data*: IL-17A is increased in several skin disorders, including psoriasis, atopic dermatitis as well as neutrophilic, granulomatous, and bullous skin diseases (30) – *Animal models*: IL-17A contributes to skin inflammation in multiple models of cutaneous inflammation, including IMQ application and K5hTGFβ1 transgenic mice (17).

Joint inflammation – *Human/patient data*: IL-17A is expressed in the synovium of RA and PsA patients and promotes bone-destructive cytokine production and bone resorption *in vitro* (28) – *Animal models*: IL-17A contributes to the immune-inflammatory events in CIA and other models of arthritis (28)

Gut inflammation – *Human/patient data*: IL17A expression is increased in inflammatory bowel disease, while neutralization of IL-17A has no effect or rather exacerbate CD (31). – *Animal models*: IL-17A neutralization exacerbates symptoms in DSS and CD4^+^ t cell transfer model of colitis, while reduced pathology in an IL-10-deficient colitogenic model. IL-17A has important roles in preserving the intestinal epithelial barrier in DSS mice (32, 33)

CNS – *Human/patient data*: IL-17A levels are increased in MS lesions and peripheral blood (34) – *Animal models*: IL-17A plays an important role in EAE (35)

Cardiovascular disease – *Human/patient data*: IL-17A/Th17 cells are increased in patients with acute coronary syndrome and correlate with systemic inflammation markers (36) – *Animal models*: IL-17A inhibition results in the reduction of the size of atherosclerotic plaques in apoE deficient mice (37)

*CNS, central nervous system; IMQ, imiquimod; RA, rheumatoid arthritis; CIA, collagen-induced arthritis; CD, Crohn’s disease; DSS, dextran-sulfate sodium; MS, multiple sclerosis; EAE, experimental autoimmune encephalomyelitis*.

The current view of the pathogenesis of psoriasis relies thus on pathogenic Th17 cells, which arise following an unknown trigger in genetically predisposed individuals as result of the production of Th17 polarizing cytokines by myeloid cells. The antimicrobial peptide LL37, in complex with nucleic acids released by dying cells, has been proposed as a possible autoantigen driving the activation of cutaneous plasmacytoid and myeloid DCs (38). Th17 cells would travel back to the skin, where they directly activate keratinocytes *via* the release of effector cytokines, among which IL-17A is the most important. Activated keratinocytes proliferate in an abnormal manner and release further inflammatory mediators and chemokines amplifying the inflammatory response (1).

**Figure 1:**
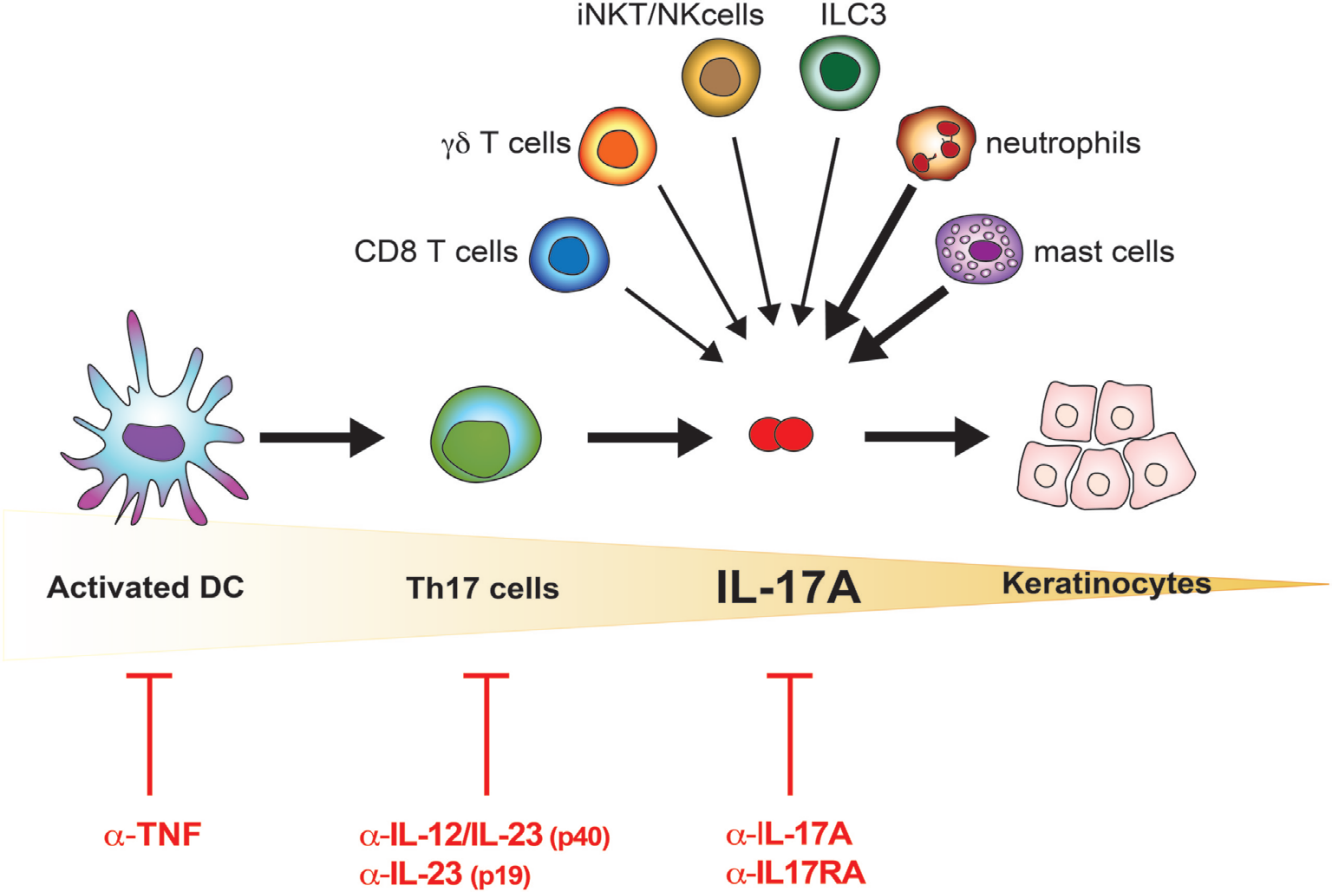
IL-17A in the pathogenesis of psoriasis. Schematic representation of the increasingly accurate selection of therapeutic targets in psoriasis. The cells possibly contributing to the bulk of IL-17A in lesional psoriatic skin are shown. Bolded arrows indicate higher contribution to IL-17A production in psoriatic lesional skin.

Recent findings provide new evidence that is slightly but definitely changing the paradigmatic view of the pathogenesis of psoriasis: from Th17- to IL-17A-driven disease (Figure 1). Reich and colleagues demonstrated that a single dose of the anti-IL-17A antibody secukinumab resulted in skin normalization as soon as 2 weeks after injection, a finding paralleled by disappearance of IL-17A + neutrophils but not T cells (39). Meanwhile, many immune cells other than Th17 lymphocytes, globally called “Type 17” cells, were reported to release IL-17A. Many of them are thymus dependent, including adaptive and natural Th17 cells, T CD8 cells, γδ T cells, and invariant NKT (iNKT) cells; others are rather thymus independent, such as group 3 innate lymphoid cells (ILC), mast cells, and neutrophils (12, 40, 41). Th17 cells, with the exception of tissue-resident memory cells, reside in lymphoid organs in steady state and drain peripheral tissues only in inflammatory situations. Conversely, the other cells are found at the periphery, particularly at mucosal and skin tissues, thus representing a potential immediate source of IL-17A. Of interest, in lesional psoriatic skin, at least from a histological point of view, IL-17A + T cells are sparse, while the bulk of IL-17A-expressing cells is represented by neutrophils and mast cells (42, 43). Whether being still debated, neutrophils and mast cells appear to actively synthetize IL-17A in the skin, and release IL-17A, at least in part, *via* extracellular trap formation (40, 42). The abovementioned subsets express RORγt and the IL-23R, and require IL-23 for their effective activation (12). This might explain why targeting specifically IL-23 through blockade of the p19 subunits represents a promising therapeutic option, even in a scenario dominated by anti-IL-17A treatments (44). IL-17A production can, however, also occur in both γδ and iNKT cells independently of IL-23 (45, 46).

The idea that psoriasis is purely a Th17 cell-dependent disease is thus replaced by the concept of psoriasis as an IL-17A-driven disease (Figure 1). This further evolution of the pathogenic concept opens new questions, which will likely allow a better understanding of the disease and a rational drug design. One of these questions is whether IL-17A has “homologous” cytokines, which would be simultaneously produced, and which might substitute for or synergize with IL-17A, or affect sites other than the skin such as the joints. In the next sections, we will thus discuss the IL-17 family of cytokines and its implication in psoriatic skin inflammation.

## An Overview of the IL-17 Family of Cytokines

IL-17A, originally termed CTLA-8, was cloned in 1993 from a rodent-activated T cell hybridoma (47). Its amino acid sequence is unusual for a cytokine, being 58% identical to the open reading frame of the T cell-tropic gammaherpesvirus *Herpesvirus samiri* (48). In the early 2000s, genomic sequencing led to the identification of several proteins structurally related to IL-17A: IL-17B, IL-17C, IL-17D, IL-17E (also called IL-25) and IL-17F. Together, these cytokines are known as the IL-17 family. IL-17F shares the highest homology with IL-17A (55%) and is often co-expressed with IL-17A (49). IL-17B, IL-17D, and IL-17C sequences overlap from 29 to 23% with IL-17A, while IL-17E appears to be the most divergent member of the family, sharing only 16% sequence homology. The members of the IL-17 family exert their functions as disulfide-linked homodimers, with a molecular weight of the monomer ranging from 17 to 21 kDa. As an exception to the rule, IL-17A and IL-17F can also form heterodimers.

**Figure 2:**
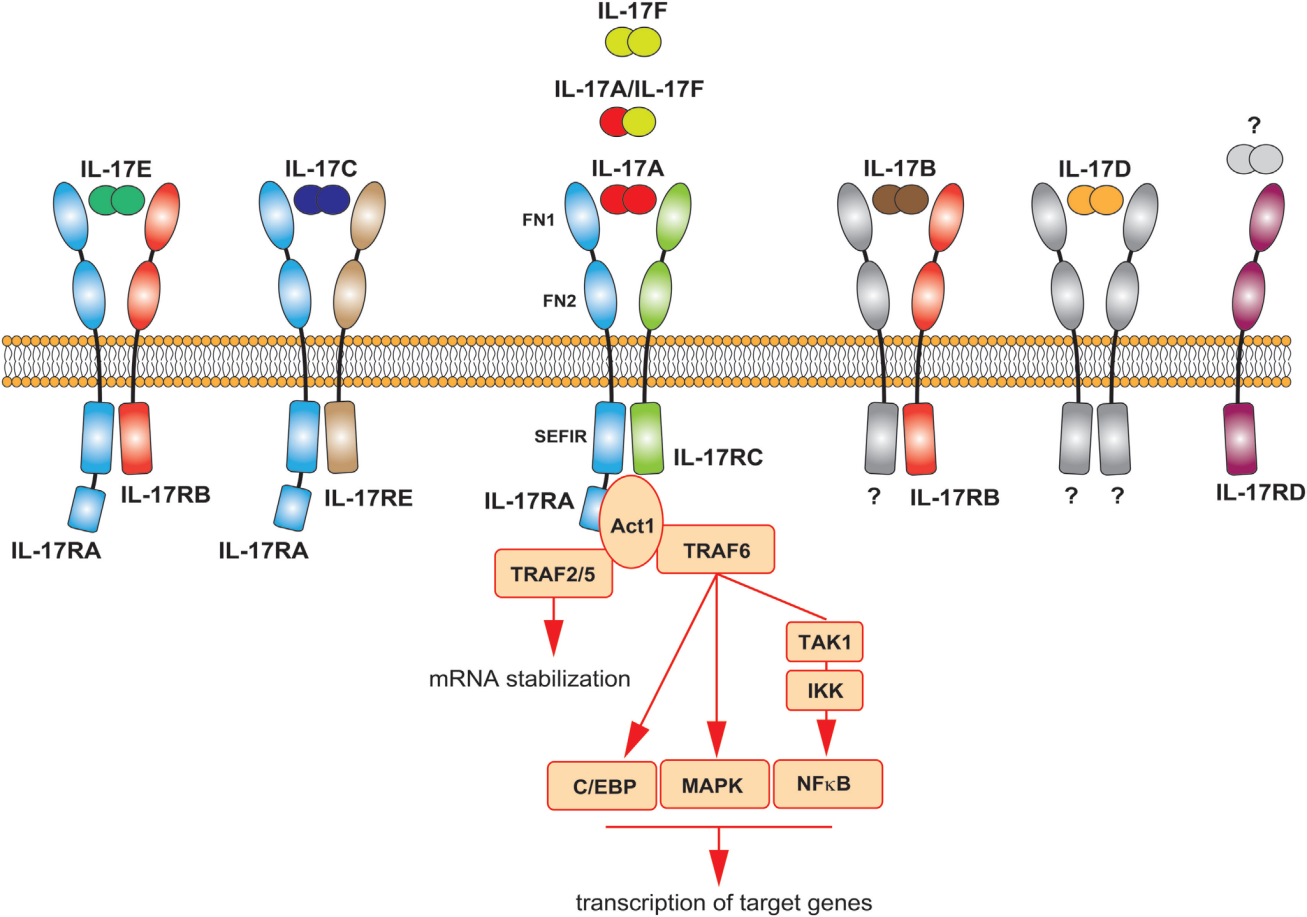
The IL-17 family of cytokines. Schematic representation of the different IL-17 cytokines and their receptors. The main signaling events downstream the activation of the IL-17A receptor are summarized.

History repeated itself for the IL-17 receptor. Discovered in 1995, the IL-17R did not fall into any previously known class of receptors (48). Later, it was discovered the existence of five homologous subunits, namely IL-17RA to IL-17RE, which together are now classified as a new class of receptors: the IL-17R family. All IL-17 cytokines signal *via* a heterodimeric receptor composed by a different combination of these subunits (Figure 2). IL-17A homodimers, IL-17F homodimers, and IL-17A/F heterodimers bind to the same receptor complex, comprising IL-17RA and IL-17RC subunits. IL-17RA is also the co-receptor used by two additional IL-17 family members; associated to IL-17RB mediates IL-17E signaling, bound to IL-17RE transduces signals by IL-17C. All subunits of the IL-17R family exhibit a broad expression pattern, with IL-17RA being ubiquitous (50).

All receptor subunits are single transmembrane chains sharing unique properties. The extracellular region of the IL-17R family members contains two fibronectin III-like (FN) domains, which mediate protein–protein interactions and ligand binding. The cytoplasmic region, at the C-terminus, contains a conserved sequence known as SEFIR (similar expression of fibroblast growth factor genes and IL-17Rs) domain. This region, which helped defining the IL-17 family as a new receptor class, is related to the TIR (toll/interleukin-1 receptor) domain found in IL-1 and toll-like receptor family. While having many features in common with the signaling induced by these innate receptors, the IL-17R-induced pathway has notable differences (12, 51). The study of the interaction of IL-17A with its receptor has generated many insights in that regard, as briefly described below and extensively discussed elsewhere (51, 52). The most striking difference is the unique usage of a cytoplasmic protein, called Act1, which also contains a SEFIR domain. Upon ligand binding, this molecule is engaged to the IL-17R complex through homotypic SEFIR interactions to mediate downstream events (53). Recruitment of Act1 represents a hallmark of IL-17 signaling, an event not shared by any other known class of receptors. Act-1 activates several independent signaling pathways operating as a docking station for different TRAF proteins. Upon recruitment of TRAF6 and its ubiquitination (Act1 may indeed function as E3 ubiquitin ligase), a cascade of molecular interactions is turned on, leading to the phosphorylation and consequent proteasomal degradation of IκB, ultimately allowing the nuclear translocation of NFκB and the activation of NFκB targeted genes (54–56). Although less clear mechanistically, TRAF6 has been also linked to the activation of MAPK pathways, including ERK, p38, and JNK, and C/EBP molecules (51). By recruiting TRAF2/5, Act-1 favors the sequestration of RNA decay factor, such as SF2 (57), as well as the activation of RNA-binding proteins as, such HuR (58), which results in increased mRNA stability of target genes. Thus, the signaling pathways downstream the IL-17A receptor may induce at least two distinct events: *de novo* inflammatory gene transcription and stabilization of target mRNA (Figure 2). Whether the other members of the IL-17 family similarly activate these pathways is unknown, but highly probable.

One peculiarity of IL-17s is that they are not strong inducers of signaling when acting in an isolated manner. Early reports have shown the implication of NFκB in mediating the pro-inflammatory role of IL-17A in fibroblasts and synoviocytes. However, IL-17A resulted to be far less potent than other inflammatory molecules, such as TNF. Nevertheless, IL-17A is an extremely powerful inducer of inflammation due to its capacity to synergistically act with other stimuli. In the case of TNF, IL-17A helps stabilizing the mRNA of the TNF-activated genes, which are inherently unstable, leading to a great amplification of TNF effects (59, 60). Therefore, even though in isolated experimental conditions, IL-17A does not appear to be potent, its physiological impact *in vivo* could be profound. An additional feature of the IL-17 family is that IL-17 cytokines might regulate several genes in a tissue-specific fashion. For instance, IL-17A was shown to induce the production of occludin specifically in the gut, where it helps maintaining the intestinal barrier integrity (46), or favor the release of kallikrein 1 by renal epithelial cells, conferring protection against candidiasis (61).

High capacity of synergy and tissue-specific functions are features shared by several members of the IL-17 family. As shown below, a considerable functional overlap, as well as main differences, characterizes the members of the IL-17 family. The following section will describe the contribution of each IL-17 family member to psoriasis, while especially focusing on isoforms other than IL-17A, as this latter has recently extensively been reviewed elsewhere (17).

## IL-17s in Psoriasis: Beyond IL-17A

### IL-17F

IL-17F, discovered in 2001 on chromosome 6p12 (the same locus as IL-17A!), is the most homologous cytokine to IL-17A and signals *via* a receptor composed by the IL-17RA and IL-17RC subunits (again, the same used by IL-17A). IL-17F levels are elevated in sera and lesional psoriatic skin compared to non-lesional tissue (62, 63). Despite that, no specific polymorphisms in the IL-17F gene have so far been associated with psoriasis susceptibility, although the IL-17F polymorphism rs763780 was linked to a better response to anti-TNF therapy (64). IL-17F is also increased in sera of atopic dermatitis patients and positively correlates with higher clinical score (65). Additional evidence of the involvement of IL-17F in psoriatic inflammation comes from experiments in mice models. Indeed, IL-17F together with IL-17A and IL-22, are rapidly induced upon imiquimod application, as result of infiltration of γδ T cells and RORγt + innate lymphocytes. Of interest, IL-17F^−/−^ mice show a higher disease resistance than IL-17A^−/−^ mice (66, 67).

Both IL-17A and IL-17F are expressed by the same immune cell types, including Th17 cells, γδ T cells and ILC3. Human *IL17A* and *IL17F* genes are found in the same locus and are genetically co-regulated, thus it is not surprising that these cytokines are often co-expressed (49). IL-23 participates in IL-17A and IL-17F co-production in Th17 cells (10). As mentioned earlier, IL-17A and IL-17F can be secreted as homodimers or IL-17A/IL-17F heterodimers. IL-17F has been shown to stimulate *in vitro* a qualitative similar pattern of genes than IL-17A, although being generally weaker, with IL-17A/IL-17F heterodimers having an intermediate potency (52). However, this is not always true, either in cell- and target-specific situations or because of synergism with other inflammatory mediators. Thus, IL-17F was shown to be more potent than IL-17A, or even TNF, to induce IL-8 and IL-6 production in normal human epidermal keratinocytes (68, 69). Or, though less potent in absolute terms, IL-17F was shown to be almost as potent as IL-17A when combined with TNF in RA synoviocytes (60, 70). Moreover, IL-17A and IL-17F have been shown to synergistically act, since their dual neutralization leads to greater downregulation of inflammatory mediators than IL-17A blockade alone in skin and joint fibroblasts (71).

With respect to their similar functions, both cytokines are needed for effective responses against mucoepithelial bacterial infections and synergistically cooperate to protect the host from fungal infections (72, 73). Consistently, inborn errors of IL-17F, as well as of IL-17RA or ACT1, display chronic mucocutaneous candidiasis (74). Candida infections are also a common adverse event observed upon anti-IL-17A or anti-IL-17RA-targeted therapies. Whether bi-specific antibodies blocking both IL-17A and IL-17F have an increased risk of Candidiasis is not yet known. Despite having similar, and sometimes even synergistic actions *in vitro*, knock-out experiments in mice revealed also diverse roles of IL-17A and IL-17F in complex *in vivo* inflammatory settings. This might well reflect tissue-specific functions and the capacity of IL-17 cytokines to synergize with other inflammatory mediators. IL-17F, at difference to IL-17A, is not required in several T-cell-dependent autoimmune diseases in mice, while being pathogenic in DSS colitis model and in acute allergic responses in the lung (72, 75–78). Nevertheless, IL-17F gene expression is increased in human active Crohn’s disease (CD) and multiple sclerosis (79, 80). Finally, IL-17F was associated with increased susceptibility in many forms of human cancer, while playing rather a protective role in colon tumorigenesis in mice (81). Table 2 shows an overview of the role of IL-17F in inflammation.

**Table 2:** Overview of the role exerted by IL-17F in inflammation.

Skin inflammation – *Human/patient data*: IL-17F is increased in psoriatic lesional skin. Elevated levels of IL-17F are found in sera from psoriatic and atopic dermatitis patients (62, 63, 65). – *Animal models*: IL-17F contributes to skin inflammation induced by IMQ application (66, 67).

Joint inflammation – *Human/patient data*: IL-17F is expressed in the synovial tissue from RA patients and contributes to human chronic joint inflammation *in vitro* and *in vivo* (62, 70, 71) – *Animal models*: IL-17F is increased in CIA (78), while playing only marginal roles on CIA and arthritis in Il1rn^−/−^ mice (72, 74).

Gut inflammation – *Human/patient data*: IL17F gene expression is increased in active CD (80) – *Animal models*: IL-17F contributes to experimental induced colitis (76, 82)

CNS – *Human/patient data*: IL-17F mRNA is increased in mononuclear cells from MS patients (79) – *Animal models*: Neutralization of IL-17F (alone) does not prevent the development of EAE (77)

Cardiovascular disease – Not investigated yet

*CNS, central nervous system; IMQ, imiquimod; RA, rheumatoid arthritis; CIA, collagen-induced arthritis; CD, Crohn’s disease; MS, multiple sclerosis; EAE, experimental autoimmune encephalomyelitis*.

In summary, IL-17F acts in a similar manner as IL-17A, although being generally weaker. IL-17F might contribute to skin manifestations and comorbidities of psoriasis in a tissue-specific fashion. The function of IL-17F may become prominent in complex cytokine milieus, and preclinical data suggest that it might be pathogenic in both joint and gut inflammation.

### IL-17B and IL-17D

Transcripts of IL-17B and IL-17D are decreased in psoriatic lesional skin when compared to non-lesional tissue (62). For that reason, they have not been studied in detail in the context of psoriasis. It is, however, relevant to briefly review their reported functions, as clinical targeting of IL-17 signaling molecules might also affect the functions of these isoforms. The roles exerted by IL-17B and IL-17D in inflammation are summarized in Tables 3 and 4, respectively.

**Table 3:** Overview of the role exerted by IL-17B in inflammation.

Skin inflammation – *Human/patient data*: IL-17B mRNA levels are decreased in lesional psoriatic skin (62)

Joint inflammation – *Human/patient data*: IL-17B is expressed in RA and OA synovia, and RA pannus (83, 84). – *Animal models*: IL-17B contributes to the exacerbation of inflammatory arthritis in CIA model (78)

Gut inflammation – *Human/patient data*: IL-17B is expressed in the intestine and stomach, unknown function (85, 86)

CNS – *Human/patient data*: IL-17B is localized to the neuronal cell bodies and axons in humans. IL-17B gene maps to a region associated with a rare autosomal recessive form of Charcot–Marie–Tooth demyelinating disease (86). – *Animal models*: IL-17B is expressed by neurons of the cerebral cortex and the adjacent hippocampal (H) layer (86).

Cardiovascular disease – Not yet investigated

*CNS, central nervous system; RA, rheumatoid arthritis; OA, osteoarthritis; CIA, collagen-induced arthritis*.

**Table 4:** Overview of the role exerted by IL-17D in inflammation.

Skin inflammation – *Human/patient data*: IL-17D mRNA levels are decreased in lesional psoriatic skin, unknown function (62)

Joint inflammation – *Human/patient data*: IL-17D is expressed in the rheumatoid nodule, unknown function (83)

Gut inflammation – Not yet investigated

CNS – *Human/patient data*: IL-17D is expressed in the brain, unknown function (87)

Cardiovascular disease – *Human/patient data*: IL-17D is expressed in the heart, unknown function (87)

*CNS, central nervous system*.

IL-17B, discovered in 2000 on chromosome 5q32, is secreted as non-covalent dimer, and signals *via* a still unknown receptor containing the subunit IL-17RB. This latter is shared with the IL-17E receptor (12). Regarding the cells producing IL-17B, information is punctual. Activated T cells do not produce IL-17B, while IL-17B was found to be expressed in neutrophils, germinal center B cells, neurons and stromal cells, and gut epithelium (88). In terms of function, high expression of IL-17B was mainly linked to poor prognosis in cancer; such as breast and gastric cancer (88) Besides that, IL-17B could play a pathogenic role in the joint (78, 83, 84). IL-17B is overexpressed in the inflammatory cartilage of the collagen-induced arthritis mouse model, where it induces the production of IL-8 and the recruitment of neutrophils. Consistently, neutralizing antibodies targeting IL-17B ameliorates signs and symptoms in this model (78). Thus, while not being involved in skin manifestation of psoriasis, IL-17B was shown to play an important role in the pathogenesis of inflammatory arthritis in preclinical models (78, 84, 89).

IL-17D, cloned in 2002 and mapping to chromosome 13q12, is the cytokine most recently added to the IL-17 family. IL-17D is most homologous to IL-17B, with 27% identity. It is secreted as disulfide-linked homodimer and signals *via* a still unknown receptor. IL-17D is found in a variety of tissues, including skeletal muscle, brain, adipose, heart, lung, and pancreas, while is poorly expressed by activated immune cells, such as lymphocytes and monocytes (87). IL-17D levels have been reported increased in rheumatoid nodules (83). Although not directly stimulating immune cells, IL-17D has been shown to modulate the production of cytokines by endothelial cells. Similar to other members of the family, these include pro-inflammatory cytokines such as IL-6, IL-8, and GM-CSF. Despite that, IL-17D demonstrates an inhibitory effect on hematopoiesis of myeloid progenitors cells *in vitro* (87). In addition, IL-17D plays a role in the control of viral infections and cancer, as IL-17D deficiency predisposes animals to these conditions (90).

Taken together, the knowledge around IL-17B and IL-17D is still limited. Overall, these isoforms induce pro-inflammatory responses in non-immune cells, leading to regulation of tumor or joint immunity, at least in the case of IL-17B. The reason of their reduced expression in psoriasis remains unknown and awaits further investigation.

### IL-17C

IL-17C was cloned at the same time as IL-17B in 2000. It maps to chromosome 16q24 and signals *via* a receptor composed by IL-17RA/IL-17RE subunits (85). IL17C shows only 23% homology with IL-17A, and unlike IL-17A, is expressed mainly by epithelial cells rather than immune cells. Much evidence links IL-17C to skin inflammation. Importantly, IL-17C is overexpressed in lesional skin of psoriatic (91, 92) and atopic dermatitis patients (93). IL-17C is secreted by epithelial cells *in vitro* in response to inflammatory stimuli, such as TNF and TLRs (91, 94, 95). Epithelial cells of the skin and the gut are also main targets of IL-17C, which acts in an autocrine manner to induce a pattern of genes similar to those induced by IL-17A, including pro-inflammatory cytokines, chemokines, and antimicrobial peptides (92, 96, 97). Similar to IL-17A, IL-17C was shown to synergize with TNF in this respect (92).

Experiments in mice confirmed that IL-17C participates in skin inflammation. Intradermal injection of IL-17C leads to epidermal thickening (96). In addition, IL-17C is upregulated in murine psoriasiform dermatitis (62, 92, 93), and IL-17C-deficient mice develop milder skin inflammation upon imiquimod application (96, 98). Conversely, selective overexpression of IL-17C in murine epidermis results in marked psoriasiform dermatitis (92). Finally, antibody-dependent blockade of IL-17C inhibited cutaneous inflammation in the IL-23-induced psoriasis model and in AD-like inflammation in mice (93).

Studies in mice also corroborate some clinical evidence obtained with targeted antibody therapies. In this respect, TNF-alpha blocking therapies result in early decrease of IL-17C levels in patients (91). The same neutralizing strategy led to an amelioration of the psoriasiform phenotype in IL-17C transgenic mice (92), pointing toward a possible role of the TNF/IL-17C axis in psoriasis. Despite this evidence, IL-17C was shown to be upregulated in paradoxical psoriasis upon anti-TNF therapy in patients presenting inflammatory bowel disease (IBD), in a mechanisms depended on IL-36γ (99). Paradoxical psoriasis onset in RA patients has also been reported after IL-6 inhibition. Similarly, genetic ablation of IL-6 leads to an increased psoriasiform phenotype in an IL-17C transgenic mouse model. This suggests that in absence of IL-6, compensatory mechanisms may occur resulting in exacerbation of disease (100).

Outside the skin, IL-17C promotes protective antimicrobial responses in the gut (96, 97, 101) and participates in mucosal responses to *Citrobacter rodentium* (97). Mice lacking IL-17C exhibited exacerbated DSS-induced colitis and IL-17C was shown to induce the production of occludin, participating in the establishment of intestinal barrier functions (101). On the other hand, IL-17C is found overexpressed in tissue from IBD patients (102, 103). In addition, IL-17C was implicated in pathogenic responses in the joints, leading to the exacerbation of arthritis induced by collagen in the mouse (78). Of interest, IL-17C was found upregulated in rheumatoid nodules and in extract from synovial fluid mononuclear cells of RA patients (83, 104). Despite its action at epithelial surface, IL-17C was shown to potentiate Th17 cell responses in EAE model (105) and to act in a pro-atherogenic manner in transgenic IL-17C mice (106, 107). With regards to cancer, IL-17C is upregulated in several forms of cancer, including colorectal and lung cancer, and was consistently shown to contribute to enhanced tumorigenesis in mice models (108). An overview of the role of IL-17C in inflammation is reported in Table 5.

**Table 5:** Overview of the role exerted by IL-17C in inflammation.

Skin inflammation – *Human/patient data*: IL-17C is increased in lesional psoriatic and atopic dermatitis skin (62, 91–93) – *Animal models*: IL-17C contributes to skin inflammation induced by IMQ application and IL-23-injection (93, 96, 98). IL-17C overexpression in keratinoctes induces psoriasiform dermatitis (92).

Joint inflammation – *Human/patient data*: IL-17C is expressed in the rheumatoid nodule (83) and by synovial fluid mononuclear cells of RA patients (104) – *Animal models*: IL-17C contributes to the exacerbation of inflammatory arthritis in CIA model (78)

Gut inflammation – *Human/patient data*: IL-17C expression is enhanced in the intestinal tissues from active IBD patients (102, 103) – *Animal models*: IL-17C participates in mucosal responses to *Citrobacter rodentium*, promotes intestinal barrier functions, and protects from DSS-induced colitis (96, 97, 101)

CNS – *Animal models*: IL-17C potentiates Th17 cell responses in EAE (105)

Cardiovascular disease – *Animal models*: IL-17C plays a pro-atherogenic role (106). IL-17C induced skin inflammation (K5-IL17C model) is associated with faster arterial thrombotic occlusion (107)

*CNS, central nervous system; IMQ, imiquimod; RA, rheumatoid arthritis; CIA, collagen-induced arthritis; IBD, inflammatory bowel disease; DSS, dextran-sulfate sodium; EAE, experimental autoimmune encephalomyelitis*.

Taken together, IL-17C appears to have much in common with the most widely studied members of the family: IL-17A and IL-17F. Synergizing with TNF, IL-17C potentiates protective antibacterial immune responses at epithelial surfaces, including the gut and the skin. Its overexpression is linked to skin conditions such as psoriasis. Similar to IL-17F, preclinical data in mice suggest that IL-17C might be pathogenic in joint disease while being protective in gut inflammation.

### IL-17E (Also Known as IL-25)

Cloned in 2001, IL-17E maps to chromosome 14q11 and signals via a heterodimeric receptor complex composed of IL-17RB (also known as IL-25R) and IL-17RA (52) (Table 6). IL-17E is more commonly known as IL-25. As for other members of the IL-17 family, IL-17E is secreted as a disulfide-linked homodimer (50), while sharing only 16% sequence homology with IL-17A. This makes IL-17E the most divergent cytokine of the family. IL-17E is produced by many cell types: including epithelial cells, endothelial cells and several immune cells, such as T cells, macrophages, type-2 myeloid cells, DC, eosinophils and ILC2s (109, 110).

**Table 6:** Overview of the role exerted by IL-17E in inflammation.

Skin inflammation – *Human/patient data*: IL-17E is increased in the lesional skin of psoriasis, atopic dermatitis, and contact dermatitis (43, 111–115)

Joint inflammation – *Human/patient data*: IL-17E is increased in the serum and synovial fluid of RA patients (116, 117) – *Animal models*: IL-17E attenuates CIA development (117)

Gut inflammation – *Human/patient data*: IL-17E is downregulated in patients with IBD (118) – *Animal models*: 17E was found to either ameliorate or aggravate colitis in mice depending on model (118, 119)

CNS – *Animal models*: IL-17E suppresses Th17 immune responses in EAE (120)

Cardiovascular disease – *Animal models*: IL-17E inhibits atherosclerosis development (121, 122)

*CNS, central nervous system; RA, rheumatoid arthritis; CIA, collagen-induced arthritis; IBD, inflammatory bowel disease; EAE, experimental autoimmune encephalomyelitis*.

Several observations argue for a possible role of IL-17E in the skin. IL-17E is upregulated in the lesional tissue of several skin inflammatory disorders: atopic dermatitis (111–113), psoriasis (43, 111, 114), and, recently, contact dermatitis (115). The precise role of IL-17E in skin inflammation may well be disease-specific. In atopic dermatitis, IL-17E is reported to negatively affect the level of the barrier protein fillagrin and to favor the loss of epidermal barrier function (111, 112). In addition, IL-17E-stimulated ILC2 cells were reported to play important roles in the regulation of skin inflammation in a mouse atopic dermatitis model (123). In psoriasis, we have found that IL-17E, produced by epidermal keratinocytes in the lesional skin, activates dermal macrophages to produce inflammatory cytokines, including TNF, and neutrophil chemokines, such as IL-8. Of note, IL-17E expression in lesional psoriatic skin correlated with the number of neutrophils, while negatively correlating with the number of T cells, suggesting that IL-17E may play a role in the chemoattraction of innate immune cells in the skin (43). These data are consistent with experiments *in vitro*, in which IL-17E has been shown to promote the expression of pro-inflammatory cytokines such as IL-8, CCL-5, and GM-CS, by human dermal and lung fibroblasts, and kidney cells (32, 78, 124, 125). The fact that a single nucleotide polymorphism (rs79877597) in the IL-17E gene associates with more severe disease and the presence of psoriatic arthritis further suggests that IL-17E may be pathogenic in psoriasis (16). In addition, improved symptoms after phototherapy was shown to correlate with a decrease in IL-17E serum levels in one patient presenting high steady-state levels of this cytokine (126). In contact dermatitis, IL-17E was shown to stimulate IL-1β production by DC, leading to enhanced Th17-, but not Th2 cell-, mediated inflammation (115).

The abovementioned results are puzzling, because the current belief depicts IL-17E as a Th2 cytokine, or a cytokine favoring Type 2 responses. IL-17E was originally reported to be expressed by Th2-polarized CD4^+^ T cells (127). Later, other immune cells were found to respond to IL-17E by producing Th2 cytokines, and transgenic overexpression or systemic administration of IL-17E in mice results in eosinophilia in addition to neutrophilia, increased production of Th2 cytokines and pathological changes in the lungs and digestive tract. These included the presence of immune infiltrates, increased mucus production, and epithelial cell hyperplasia (128, 129). IL-17E was found to induce Th2 cell differentiation and activation (110), being thus crucial in host immune responses to nematode infections (130) and the development of allergic airway inflammation (131). Furthermore, IL-17E was shown to suppress Th17-mediated autoimmune diseases in mice, such as EAE and rheumatoid arthritis, mainly by skewing the immune system toward a Th2 response (120). In the gut, IL-17E may play both anti- and pro-inflammatory roles depending on the type of inflammation that is present (118). Moreover, IL-17E has been shown to inhibit atherosclerosis development in mice (121).

In summary, IL-17E is essential for protection against parasites and plays critical roles in Th2-mediated diseases (such as allergic asthma). In addition, it may limit Th17 cell responses, at least in mouse models of EAE and colitis. On the other hand, IL-17E is pathogenic in skin diseases, where it may function in « opposite » fashion and rather favor Th17 responses and recruitment of neutrophils. Thus, IL-17E effects appear to be highly tissue-specific.

## Clinical Implications

Neutralization of IL-17A (secukinumab and ixekizumab) or the receptor subunit IL17RA (brodalumab) *via* monoclonal antibodies represents a highly effective approach to treat psoriasis (26, 27, 132). Blockade of IL-17RA also results in the inhibition of an array of members of the IL-17 family: namely IL-17A, IL-17F, IL-17C, and IL-17E. In addition to these licensed drugs, several other molecules targeting multiple IL-17 family members [IL-17A and IL-17F in the case of bimekizumab (71)], or the IL-17 pathway (either upstream, i.e., IL-23, or downstream signaling molecules) are in clinical development (133). Thus, a better knowledge of the structure of the IL-17 family and the function of their members with respect to inflammation is critical.

Although direct comparative trials have not been performed yet, indirect evidence suggests that IL-17RA inhibition may be superior to IL-17A inhibition, at least with respect to a greater likelihood of achieving PASI 100 and PASI 90 (134). Consistently, IL-17A, IL-17F, IL-17C, and IL-17E (all signaling *via* IL-17RA) have similar pro-inflammatory functions in the skin and have shown to play a pathogenic role in psoriatic skin manifestations. However, these same cytokines have also been reported to have non-redundant functions outside the skin, at least in the mouse. Thus, IL-17E has a protective role in CNS inflammation (120) and participates in protective responses against parasites in the intestine (130, 135, 136); and IL-17F and IL-17C are important for antibacterial immunity at epithelial surfaces (72, 73, 96, 97, 101). It remains to be addressed whether these tissue-specific functions exist in man too, and whether neutralization of several IL-17 cytokines might generate unwanted side effects outside the skin.

The possibility of inhibiting more than one IL-17 family member at a time could, however, reveal to be promising, as shown in the case of bimekizumab, a bi-specific anti-IL-17A and anti-IL-17F antibody (71, 137). While having a similar efficacy in the skin compared to IL-17A inhibition, this approach seems to perform better at the level of the joints, though larger trials have still to confirm this initial observation. This is consistent with a pathogenic role of IL-17F in joint inflammation, as observed in the mouse. Since IL-17C also participates in joint damage in mouse models of arthritis (133), it remains to be addressed whether its inhibition could be advantageous. On the other hand, both IL-17F and IL-17C are protective in murine gut inflammation (76, 96, 97, 101, 138). Whether a higher risk of intestinal inflammation due to IL-17F or IL-17C inhibition exists is unknown.

In summary, IL-17 family cytokines may elicit similar effects in target cells, but simultaneously may have very different (and sometimes opposite) functions in a tissue-specific manner. In addition, IL-17 cytokines have a great capacity of synergism, and their potency could be highly augmented by the cytokine milieu found at the inflamed site. These properties of the IL-17 family have direct clinical implications, as blocking more than one cytokine is a strategy currently under evaluation. A more comprehensive understanding of the mechanisms orchestrating the tissue-specific functions of the IL-17 family members in humans, and the relationship (causal or effector) existing among the different members of the family is required for a more rational drug design.

## Author Contributions

NB and W-HB defined the content of the manuscript, contributed to literature search and manuscript writing. LS contributed to literature search and manuscript writing. NB created graphical illustrations. All authors approved the final version of the manuscript.

## Conflict of Interest Statement

NB and W-HB have received a research grant to study the role of Janus kinases in the pathogenesis of psoriasis from Pfizer. W-HB received honoraria as a speaker or advisor from the following companies: Abbvie, Almirall, BMS, Celgene, Janssen, Leo, Lilly, Novartis, Sun Pharmaceuticals, UCB. LS has no conflict of interest to declare.
